# RNA Origami: Packaging a Segmented Genome in Orbivirus Assembly and Replication

**DOI:** 10.3390/v13091841

**Published:** 2021-09-15

**Authors:** Po-Yu Sung, Polly Roy

**Affiliations:** Department of Pathogen Molecular Biology, Faculty of Infectious and Tropical Diseases, London School of Hygiene and Tropical Medicine, Keppel Street, London WC1E 7HT, UK; po-yu.sung@lshtm.ac.uk

**Keywords:** *Orbivirus*, RNA packaging, segmented genome virus

## Abstract

Understanding how viruses with multi-segmented genomes incorporate one copy of each segment into their capsids remains an intriguing question. Here, we review our recent progress and describe the advancements made in understanding the genome packaging mechanism of a model nonenveloped virus, Bluetongue virus (BTV), with a 10-segment (S1–S10) double-strand RNA (dsRNA) genome. BTV (multiple serotypes), a member of the *Orbivirus* genus in the *Reoviridae* family, is a notable pathogen for livestock and is responsible for significant economic losses worldwide. This has enabled the creation of an extensive set of reagents and assays, including reverse genetics, cell-free RNA packaging, and bespoke bioinformatics approaches, which can be directed to address the packaging question. Our studies have shown that (i) UTRs enable the conformation of each segment necessary for the next level of RNA–RNA interaction; (ii) a specific order of intersegment interactions leads to a complex RNA network containing all the active components in sorting and packaging; (iii) networked segments are recruited into nascent assembling capsids; and (iv) select capsid proteins might be involved in the packaging process. The key features of genome packaging mechanisms for BTV and related dsRNA viruses are novel and open up new avenues of potential intervention.

## 1. Introduction

Genome packaging is an essential process in the virus life cycle, as at least one genome (or one set of RNAs of a multi-segmented genome) must be incorporated into the limited space available in the capsid. Two strategies are used by viruses for genome packaging; one is the assembly/recruitment of the genome prior to capsid formation, and the other is the formation of empty protein capsids into which the genome is packaged. The latter strategy is particularly notable in the double-stranded DNA (dsDNA) viruses, such as tailed bacteriophages and herpesviruses, and the double-stranded RNA (dsRNA) viruses (e.g., φ6 bacteriophage) [1,2,3]. For either approach, packaging signals are usually required to distinguish the viral genome from cellular nucleic acids.

For viruses with multi-segmented genomes, such as members of the *Orthomyxoviridae* (influenza), *Phenuiviridae* (e.g., Rift Valley Fever Virus), *Arenaviridae* (e.g., Lassa Fever Virus), *Birnaviridae* (e.g., Infectious bursal disease virus), *Hantavirus*, *Nairovirus*, *Herbevirus*, and *Reoviridae* (e.g., Bluetongue virus and Rotavirus), the virus not only needs to selectively package viral RNAs but must also ensure that at least one complete set of genomic segments is recruited within each capsid. With viruses where the genomic segment number is low, for example the two-segmented (*Birnaviridae*) or three-segmented (*Phenuiviridae*) genome viruses, the packaging of segments appears to be stochastic with a significant portion of virus particles containing an incomplete genome complement [4,5]. However, this becomes mathematically impossible for more complex viruses, as the chance of producing infectious particles from a random packaging mechanism would be too small to ensure survival. For example, most of the *Reoviridae*, which includes many medically important members, contain nine to 12 genome segments, and studies have shown that the particle to infectivity ratio is low, suggesting that a selective packaging mechanism must operate [6,7,8]. The genome packaging mechanism of the 10-segmented Bluetongue virus (BTV), in the *Orbivirus* genus within the *Reoviridae* family, has been studied extensively as a representative of this large family of multi-segmented viruses. Here, we summarize our current understanding of the packaging mechanisms utilized by BTV and related viruses.

## 2. Overview of Orbivirus Replication

**Figure 1:**
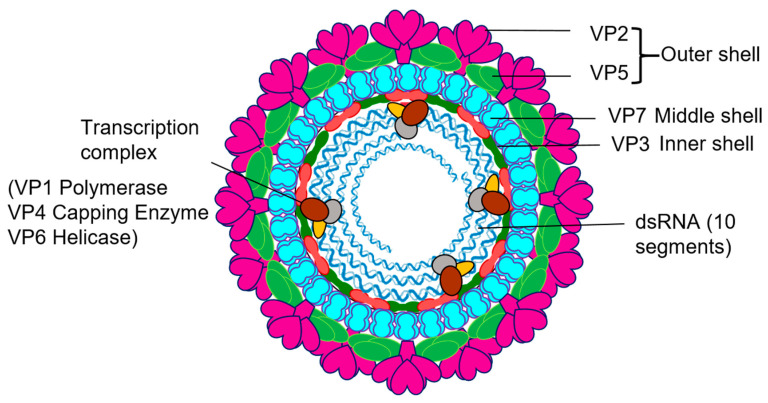
*Orbivirus* particles and components. Schematic diagram of *Orbivirus* showing the positions and structural organization of its components.

The *Orbivirus* genus consists of several major animal pathogens, including the viruses that cause high morbidity and mortality BTV, African Horse Sickness Virus (AHSV) and Epizootic haemorrhagic disease virus (EHDV), and it is one of the 15 genera of *Reoviridae* with a large number of members recognised to date. A distinctive feature of members of this family is their multipartite dsRNA genomes. BTV and other Orbiviruses are transmitted by a range of hematophagous arthropod vectors, most replicating both in insect and mammalian cells, and they are an economically important group that infects a wide range of hosts, often causing serious disease in animals. Orbiviruses are structurally complex with a genome of ten dsRNA segments (S1–S10) enclosed by a four-layered protein capsid [9,10,11] (Figure 1). The outer capsid is removed shortly after cell entry to release the inner capsid, the ‘core’, into the cytoplasm [11,12,13,14]. The core is composed of two major proteins, VP7 and VP3, and three minor proteins that make up the transcription complex (TC), including VP1 polymerase, VP4 capping enzyme, and an RNA binding/putative helicase and RNA packaging protein VP6 [15,16,17,18,19,20]. Within the core, the TC repeatedly transcribes the ten genomic dsRNA segments and the ssRNA transcripts extrude from the core to serve as messengers for viral protein synthesis and nascent dsRNA segment synthesis, thereby initiating virus replication. These steps require efficient co-ordination between the full complement of genomic segments and the resident enzyme activities.

**Figure 2:**
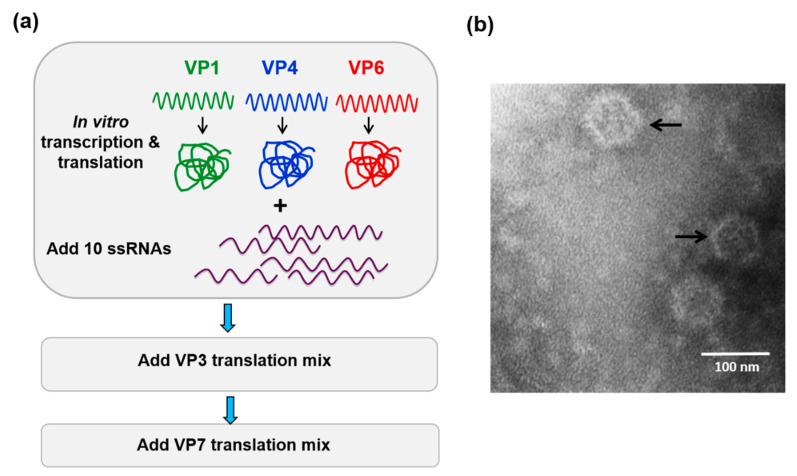
In vitro cell-free assembly assay reconstitutes BTV cores. (**a**) Cartoon of the cell-free assembly assay of BTV cores showing the sequential inclusion and incubation of BTV proteins and 10 ssRNA transcripts. (**b**) Electron micrograph of reconstituted BTV cores (indicated by arrows) [21].

A unique cell-free assembly (CFA) assay has shown that the 10 positive sense ssRNA segments (+ssRNAs) of BTV associate with the three proteins of the TC prior to encapsidation by VP3 (to form a subcore particle), followed by the addition of the VP7 layer leading to the formation of a stable core [21] (Figure 2). Then, these packaged ssRNAs serve as templates for dsRNA synthesis, resulting in an equimolar set of all genome segments. Importantly, these in vitro assembled cores, with a complete set of genomic RNA segments, are replication competent, but how the 10 ssRNAs are recognised precisely and packaged with the correct stoichiometry has been unclear until recently. However, use of the CFA assay together with BTV reverse genetics [22] and other newly developed assay systems has now uncovered key steps of the segmented RNA genome packaging process and the virus assembly that follows. 

## 3. Structural Constraints in Genomic Segments Regulate BTV Genome Packaging 

In vitro assembly studies demonstrated that the sorting and packaging of viral RNA segments occur at the positive sense ssRNA level. In order to ensure that every progeny virion contains a complete set of 10 genomic segments, sorting and packaging must be highly specific and regulated. The 10 genomic BTV segments possess variable numbers of nucleotides (822–3944 bp), although both termini of each segment have a highly conserved complementary five or six-nucleotide sequence. Furthermore, the length of each segment is more or less conserved among the different BTV serotypes [23,24]. The length of the 3′ untranslated region (UTR) of each segment differs among the 10 segments but is highly conserved among the same segments of each serotype, leading to the hypothesis that the 3′ UTR may play a role in RNA packaging. 

**Figure 3:**
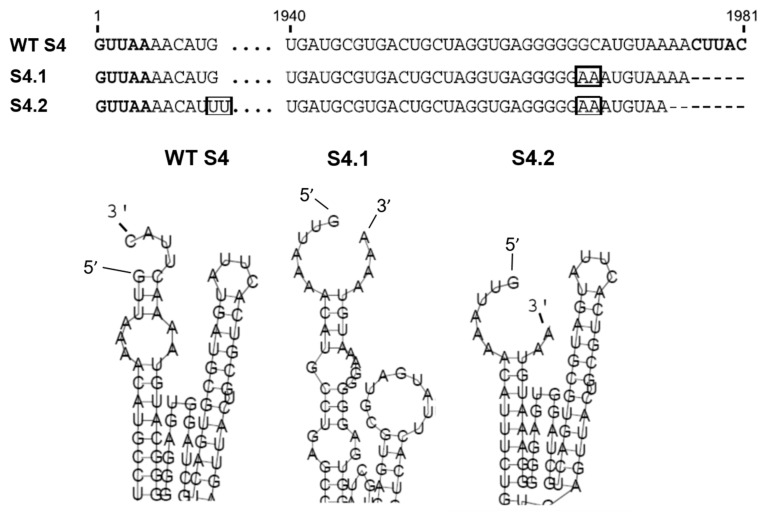
BTV S4 UTRs and secondary structure and effects of mutations. Upper panel: 5′ and 3′ UTRs sequences of BTV-9 wild type (WT) S4, mutant S4.1 and S4.2. Boxes show the nucleotides pairing which rescues loop-like structure. Lower panel: Detail of the secondary ssRNA structural changes of S4.1 and S4.2 as predicted using RNA fold prediction software [25].

Within the same Orbivirus species, different serotypes can exchange genome segments without affecting virus replication, for example, segment S4 (S4) of BTV-1 and BTV-9 are exchangeable either by coinfection or by a combination of infection and RNA transfection, and this property was used to assess the role of the 3′ UTR in RNA packaging. A series of mutations in the S4 segment of BTV-9 was made, and their capacity to be packaged into BTV-1 particles was assessed [25]. As predicted, the full-length S4 of BTV-9 was packaged successfully into BTV-1 cores, but deletion of the complete 5′ UTR or 3′ UTR, with or without the conserved regions in S4, prevented packaging. Secondary structure analysis of S4 of BTV-9 using RNAfold predicted an interaction between the 5′ and 3′ complementary hexanucleotides to form a structure composed of a hairpin loop and a stem-loop separated by a potentially flexible stretch of nucleotides (Figure 3) [25]. Subsequent in vivo packaging assays confirmed that when the mutations did not alter the predicted secondary RNA structure, the mutant S4 could be packaged, but that mutant RNAs in which the secondary structure was abolished failed to package (Figure 3) [25]. Furthermore, a chimeric segment that maintained the same putative conformational structures but contained unrelated internal sequences was packaged successfully, confirming that the UTR resident structural elements are involved in RNA segment packaging.

## 4. How Is a Complete Set of ssRNA Molecules Selected for Packaging? 

**Figure 4:**
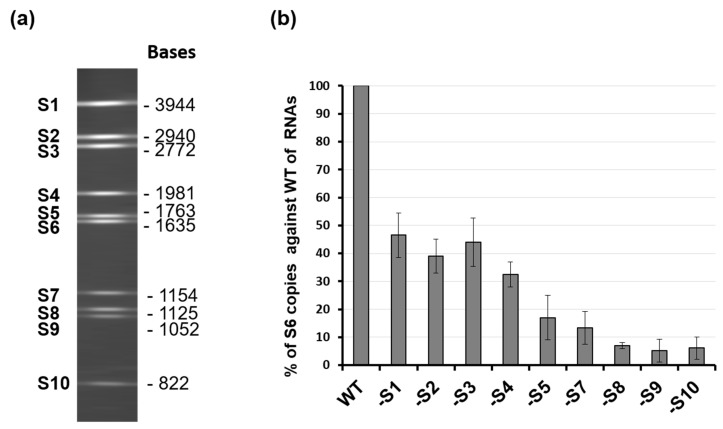
Exclusion of specific BTV RNA segment influences genome packaging. (**a**) 10 genomic RNA segments of BTV-1 and their sizes. (**b**) Quantification of the effect of segment exclusion. Ten BTV ssRNAs (WT) or ssRNAs excluding one ssRNA at a time (S1, S2, etc.) were used in the CFA assay. Packaged ssRNA was compared with WT control in the same experiment, and packaging efficiency was calculated [26].

While it was clear that the secondary structure of each segment, driven by the 5′ and 3′ UTR, was essential for the packaging of that specific segment, the previous study did not explain the packaging process, allowing the coordinated selection of all 10 genome segments successfully. It was noted that the complexity of multi-segmented genome packaging could be reduced if packaging was restricted to only some segments, with the others drawn in via their interaction with those that package, essentially a ‘follow-the-leader’ model of genome incorporation. Such a hypothesis could only be proven by an appropriate in vitro RNA packaging system, such as cell-free BTV CFA assay described (see Figure 2, [21]). Using this system, it was possible to show, for the first time, that there is a packaging order for BTV ssRNA segments. By excluding one segment of the set of ten segments, one at a time, it was shown that the smaller segments (S7–S10), but not the larger segments (S1–S4), are essential for packaging of the complete set of BTV genome segments (Figure 4) [26]. The exclusion of S1, S2, S3, or S4 all together only had a moderate impact on packaging the rest of the genome. The data clearly indicated that BTV genome segments are not packaged individually but rather through certain interacting networks among different segments, and the networking most probably starts with the smaller segments. Replacing or deleting the UTRs of the smallest genome segment, S10, showed a drastic effect on the packaging of the complete set of genome segments supporting not only “the follow-the-leader” model of genome incorporation but also reiterating the importance of UTRs in packaging.

**Figure 5:**
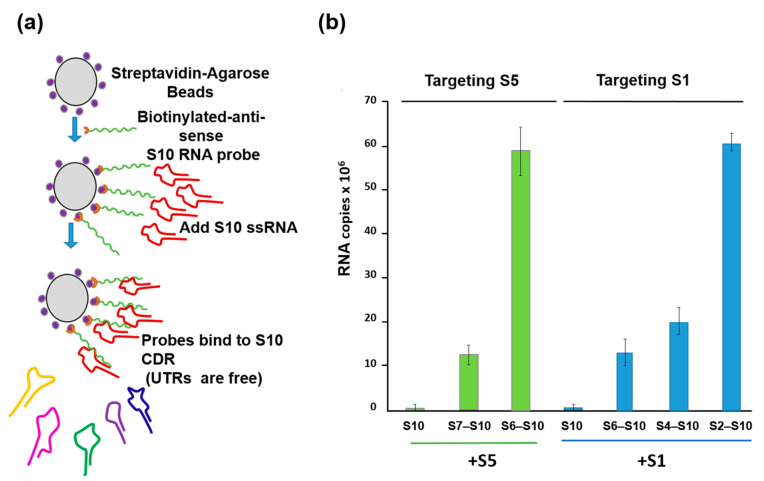
RNA beads assay demonstrating the interactions between S10 and smaller segments followed by the larger segments in a specific order. (**a**) A schematic for RNA–RNA interaction assay based on BTV S10-coated beads. (**b**) S5 (**left**) and S1 (**right**) need intermediate segments to interact with S10. Left panel: BTV-1 S5 was incubated with S10 beads alone (S10) or with a mixture of S7 to S9 (S7–S10) or S6 to S9 (S6–S10). Right panel: BTV-1 S1 was incubated with S10 beads alone (S10) or with a mixture containing S6 to S9 (S6–S10), or S4 to S9 (S4–S10), or S2 to S9 (S2–S10). UTRs: Untranslated regions. CDR: Coding region [26].

To substantiate this model further, a second in vitro ssRNA interaction assay based on a biotinylated primer-coated streptavidin bead was developed, which did not require the presence of any viral protein (Figure 5a). Using this assay, the smaller segments, particularly the smallest BTV RNA segment (S10), with an unusually long (113 bp) 3′UTR, had a high affinity for the three other small ssRNAs (S7–S9) but not for the larger segments or for the unrelated rotavirus segments [26]. Moreover, the data suggested that there is an order of RNA–RNA interaction and that complex formation among the 10 segments starts with the smaller segments (S7–S10), which form a complex capable of binding the medium segments (S4–S6), which in turn binds the larger ones (S1–S3) (Figure 5b). Then, this RNA complex is packaged into capsids possibly through protein–RNA interaction. 

These data suggest that the genome segments may be pre-assembled prior to being packaged into the capsid and that this assembly is likely based on a network of segments, which is initiated by the smaller segments. 

## 5. RNA Complex Formation and RNA Network May Initiate Packaging 

Complementarity between the segments means that the virus packages a reduced number of segment complexes rather than each genomic RNA segment individually. The concept of RNA–RNA interactions as specifiers of biological choice has emerged as a major factor for many non-coding RNAs, and its understanding underpins many RNA directed events including transcription, splicing, translation, and RNA decay [27,28,29]. In other RNA viruses with segmented genomes (e.g., Influenza A virus, Red clover necrotic mosaic virus), *trans*-acting RNA–RNA interactions have also been found to play an important role in genome packaging [30,31]. 

**Figure 6:**
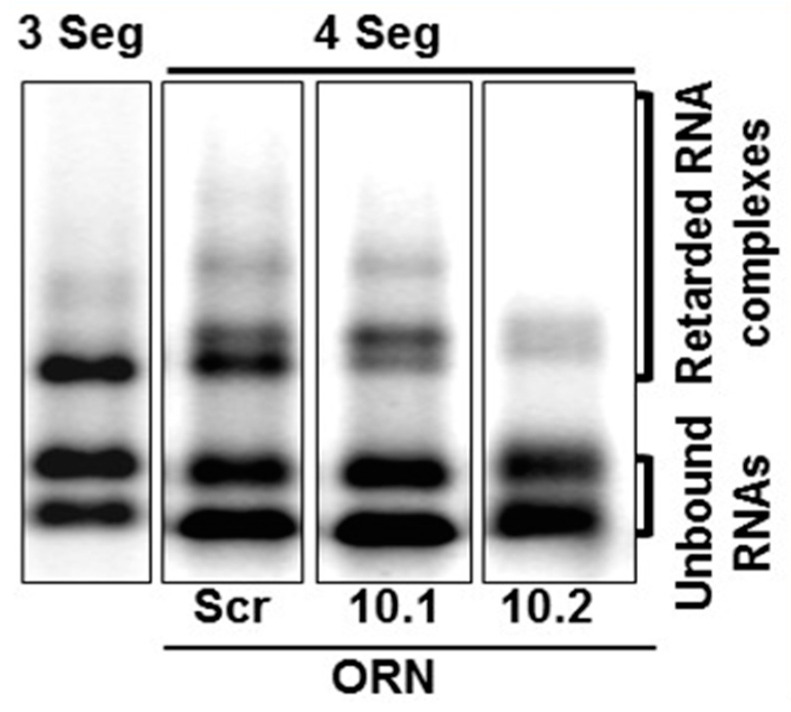
EMSA shows that BTV segments form RNA complexes. S8–S10 (3 Seg) or S7–S10 (4 Seg) RNA complexes are shown in agarose gel. Oligoribonucleotides (ORNs) targeting S10 (S10.1 and S10.2) or scrambled sequence (Scr) have different impact on RNA complex formation [32].

**Figure 7:**
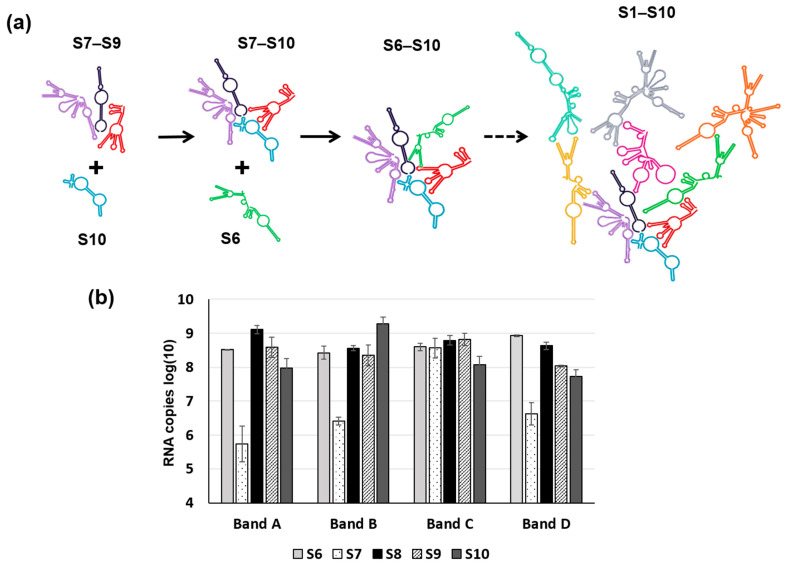
A Schematic of RNA–RNA interaction during packaging. (**a**) A schematic shows three stages of sequential RNA–RNA interactions of BTV ssRNA in stages, from the smaller RNA segment complex S7–S9 to the complete set of S1–S10 complex. S10: blue, S7–S9: purple, black, red and S6: green. (**b**) Combination of S6–S10 (each segment indicated below the char) produces four sizes of RNA complexes (Band A–D) in agarose gel by EMSA and the RNA composition of each band analysed by qRT-PCR. For details, please read reference [35].

RNA complex formation by the smaller segments (S7–S10) of Orbiviruses has been visualised by a modified electrophoretic mobility shift assay (EMSA). Multiple complexes with different conformations could be visualised in agarose gels when three or four RNA segments were co-transcribed together (Figure 6) [32]. Moreover, as the S10 3′ UTR was already shown to be important for RNA packaging and virus replication, short antisense nuclease-resistant oligo-ribonucleotides (ORNs) complementary to the 3′UTR of S10 were shown to disrupt complex formation and also to inhibit their packaging in the CFA. The same ORNs inhibited virus replication in cell culture, linking the in vitro observations of RNA complex formation with a virus phenotype (Figure 6). Similarly, mutations in the S10 3′UTR that inhibited complex formation failed to recover viable viruses by the BTV reverse genetics system [22,32]. These data provide direct evidence that the two processes, RNA complex formation and RNA packaging, are linked, and similar results have since been obtained with other Orbiviruses as well as with rotaviruses [30,33,34], suggesting that this could be a common mechanism shared by all *Reoviridae* members. However, although these studies indicate a sequence specific interaction that is key to genome packaging, the nature of the interaction and how it is involved in complex formation was still lacking. To address this, we developed a dynamic network approach for the modelling of BTV segment association focusing on different stages of RNA segment assembly, reassessing the interaction probabilities at each stage of complex formation for the five (one medium and four smaller) BTV segments (S6–S10) [35]. The computer-generated model identified that interactions among S6–S10 occur at three stages of the complex formation: the initial S7+S8+S9 complex (Stage 1), its association with S10 (Stage 2), and its association with S6 (Stage 3) [35]. Both interacting sites and the geometries of the Stage 2 and 3 complexes need to change, allowing the incoming segments to join the growing complex (Figure 7a). Furthermore, interactions between segments occur at multiple specific sites, dispersed across each segment. As a proof-of-concept of the predicted model, S6–S10 segments were co-synthesized, followed by EMSA, which exhibited a set of four distinct sized complexes. Each of these complexes was found to be assemblies of all five RNA segments but in different molar ratios, indicating that each existed in a different conformation (Figure 7b). EMSA data were further supported by the isolation of the five segments from a single band using velocity gradient ultracentrifugation. Furthermore, probing five of the contact sites between S6 and S10 by mutagenesis confirmed a role in segment assortment and complex formation as they failed in virus recovery by reverse genetics [35]. This interdisciplinary approach yielded two important conclusions: (i) RNA–RNA contacts occur widely across the segments, and (ii) it is possible to identify the contacts that play essential roles in RNA complex formation, thus providing a hierarchy among contacts. 

These proof-of-principle data demonstrate a new concept for how multipartite genomic RNA segments might be combined prior to, or during, genome packaging. Thus, there is a unique opportunity to explore exactly how such RNA networks are formed and what role they play in genome recruitment into nascent assembling capsids in other Reoviruses such as Rotavirus.

## 6. Role of Viral Inner Capsid Proteins in RNA Packaging

Although the structural conformation of each RNA segment and RNA–RNA interactions between segments play pivotal roles in regulating BTV genome sorting and packaging, viral proteins may also be involved in this process. Our current understanding is that the 10 positive-sense ssRNA segments of BTV first associate with the TC prior to encapsidation by the VP3 layer of 10 decamers, resulting in the subcore particle. Of these four proteins, VP6 and VP3 appear to play key roles in genome recruitment and localization in the assembling capsid.

**Figure 8:**
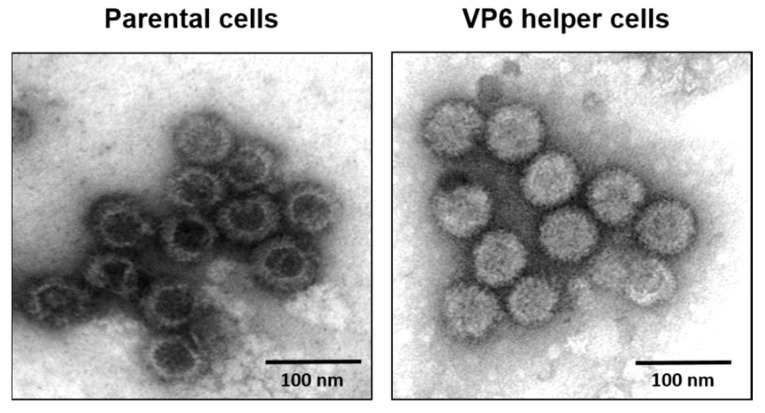
Electron micrograph of BTV core particles. Left: Purified cores from VP6-deficient parental cells showing empty particles. Right: Purified cores from VP6 helper cells showing complete cores particles similar to the native cores [16].

The smallest structural protein (329 aa), VP6, is unique for the *Orbivirus* genus in the *Reoviridae*. This internal core protein has a strong affinity for both ssRNA and dsRNA molecules, suggesting that it is closely associated with the viral genome [36]. VP6 has also been suggested to be an RNA helicase, despite poor homology with known helicases [19,37]. Virus recovery by reverse genetics has confirmed that VP6 is essential for BTV replication and that modified BTV strains lacking VP6 do not replicate in normal cells but only in a VP6 helper cell line [38]. Furthermore, when VP6-deficient viruses were grown in VP6 helper cells and used for the infection of normal cells, viral proteins were synthesized and assembled as empty particles without the viral genome. These data suggest that VP6 may be responsible for genome recruitment (Figure 8) [16]. 

**Figure 9:**
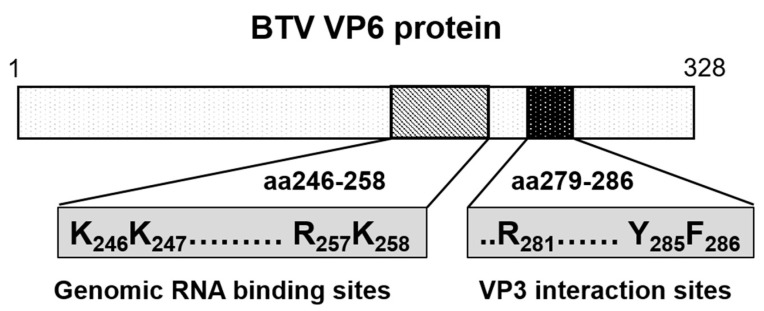
Diagram showing the genomic RNA-binding sites and the VP3 interacting sites in VP6 protein. Genomic viral RNA-binding region (aa 246–258) and capsid protein VP3 binding region (aa 279–286) of VP6 are shown in diagram. Residues critical for viral replication are indicated.

A recent proteomic approach (RNA-cross-linking and peptide fingerprinting, RCAP) has allowed the identification of RNA-binding sites in VP6 in two different scenarios: (1) BTV ssRNA complexed with recombinant VP6 (reVP6) and (2) genomic dsRNA with VP6 in association with VP3 within the purified BTV core. The data revealed that both reVP6 and capsid-associated VP6 (caVP6) have multiple distinct RNA-binding regions. Three regions of reVP6 were strongly associated with the binding of ssRNA, aa 2–15, aa 110–141, and aa 220–284. However, when the positively charged residues in these three regions were mutated and tested for virus recovery, mutations in the first two sites had no effect, suggesting they are not critical for virus replication. In contrast, mutation of the third site prevented virus recovery, indicating that only this site has specificity for viral ssRNA binding. The same site was also found to be critical for RNA binding by caVP6. Further in vitro and in vivo studies confirmed that a motif within this shared binding region preferentially binds BTV ssRNAs over non-BTV ssRNAs shown by an RNA-binding completion assay and is essential for RNA packaging (Figure 9) [20]. While these data suggest that VP6 acts as an RNA chaperone protein, the precise RNA sequence(s) that VP6 binds to for packaging and whether VP6 is the only active participant in RNA recruitment by the core still remain unknown. 

**Figure 10:**
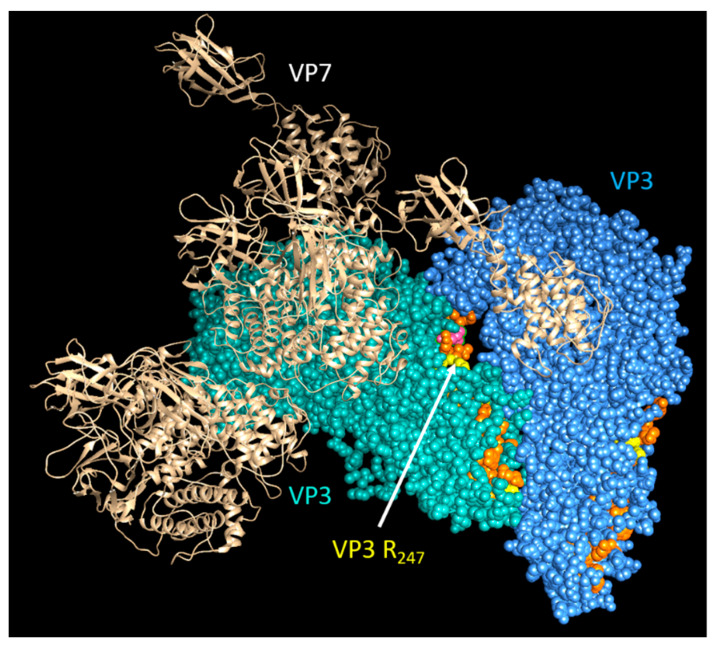
Predicted structural location of genomic RNA-binding sites on VP3 within the core. A cartoon showing the view of a section of the BTV core looking through inward: cyan and blue VP3 molecules indicate VP3 dimer of BTV core; the RNA-interacting sites are shown in orange and yellow.

Cryo-EM and asymmetric reconstructions of the BTV vertex have allowed obtaining an atomic model of the large polymerase protein VP1, but not VP6, suggesting that it may be either too flexible to obtain a location or masked by genomic RNA [39]. However, recent molecular studies have confirmed that VP6 directly interacts with VP3 via its C-terminal end and that the perturbation of this interaction abolishes RNA packaging [17]. Therefore, it is hypothesized that VP6 is responsible for recruiting the RNA complex(s) to the inner capsid protein VP3 via its specific VP3 binding residues aa 281, aa 285, and aa 286, which are found downstream of its RNA-binding site (Figure 9) [17]. Thus, VP3 itself may also have a role in RNA packaging. Therefore, mapping the RNA-binding sites of VP3 could provide further insights into RNA packaging and viral replication. Preliminary RCAP analysis of the purified core has identified three RNA-binding regions within VP3 (aa 245–253, aa 499–511 and aa 517–536), of which the aa245–253 site is located adjacent to a channel in the capsid shell through which ssRNA has been suggested to exit (Figure 10). A functional role for aa 245–253 in RNA egress from the core might be consistent with the other two suggested RNA-binding regions playing a role in genome packaging, although confirmation of this will require further experimentation.

## 7. Do Any of the BTV Non-Structural Proteins Play Any Role in RNA Packaging?

Various in vitro assays have demonstrated that BTV non-structural proteins are not directly involved in RNA sorting, recruiting, or packaging. However, the phosphorylated non-structural protein 2 (NS2) of Orbiviruses has a high affinity for viral ssRNA over host RNAs, and phosphorylation is not necessary for the RNA-binding properties [14,40]. NS2 is an essential component of the virus primary replication complex, together with four subcore proteins, VP1, VP3, VP4, and VP6 in infected cells, although it is not required in the in vitro CFA assay [21]. Similar functional activities are shared by rotavirus NSP2 [41,42]. In vitro, BTV NS2 interacts with different RNA segments via several distinct RNA structures undoubtedly to discriminate viral from cellular RNAs within infected cells [43]. Furthermore, NS2 recognizes specific regions within each BTV segment via specific secondary structure and the binding of two different viral RNAs can occur independently. Chemical and enzymatic probing of these predicted secondary structures, together with mutations that disrupted the structures, confirmed that each RNA possesses a distinct secondary structure that is recognized by NS2. These data imply that NS2 may recruit virus ssRNAs selectively from other RNA species within the infected cytosol during virus replication [44]. However, since BTV structural proteins and RNA segments alone can assemble to form infectious particles in the absence of the NS2, it must play a role in vivo as a condenser of the necessary components, RNA and protein, required for assembly in the host cytoplasm. The detailed role of NS2 remains to be explored.

## 8. Concluding Remarks

We have summarized how multipartite genomic ssRNA segments are combined prior to, or during, packaging using an experimental model virus, BTV, with direct implications for related medically important viruses (e.g., Rotaviruses) and many other complex dsRNA viruses that infect human, animals, and plants. An extensive background knowledge coupled with multidisciplinary approaches, including reverse genetics and a cell-free RNA packaging assay, have facilitated our understanding of the process. It includes the dynamic nature of the segmented RNA genome, the importance of the UTRs for driving the conformations of each RNA necessary for the next level of interaction, how exactly such RNA networks are formed, how they are recruited into nascent assembling capsids, and what capsid proteins might be involved. However, certain key questions are still not clear. For example, although it is evident that RNA–RNA interactions are based on complementary sequences, the precise sorting signals required for BTV RNA packaging have not been identified. 

Nevertheless, the current studies have uncovered a completely new concept of packaging in which a reduced number of RNA segment complexes is packaged rather than each segment individually. Such fundamental mechanisms of RNA network formation by multiple segmented genomes offer new possibilities for future antiviral design which block this essential and conserved process for BTV and related pathogenic viruses of humans and animals or for designing defective interfering particles containing an incomplete set of genomic segments that may act as effective vaccines.

## Data Availability

The data that support the findings of this study are available from the corresponding author, P.R., upon reasonable request.
